# Feasibility of telepsychology support for patients with advanced cardiorespiratory diseases and their caregivers

**DOI:** 10.3389/fpsyg.2022.909417

**Published:** 2022-08-10

**Authors:** Lidia Gazzi, Laura Comini, Simonetta Scalvini, Irene Taccolini, Michele Vitacca

**Affiliations:** ^1^Psychology Service, Neurorehabilitation Unit of the Institute of Lumezzane, Istituti Clinici Scientifici Maugeri IRCCS, Brescia, Italy; ^2^Scientific Direction of the Institute of Lumezzane, Istituti Clinici Scientifici Maugeri IRCCS, Brescia, Italy; ^3^Cardiac Rehabilitation and Continuity Care Unit and Telemedicine Service of the Institute of Lumezzane, Istituti Clinici Scientifici Maugeri IRCCS, Brescia, Italy; ^4^Respiratory Rehabilitation of the Institute of Lumezzane, Istituti Clinici Scientifici Maugeri IRCCS, Brescia, Italy

**Keywords:** COPD, cardiorespiratory, rehabilitation, heart failure, telepsychology

## Abstract

**Objective:**

The aim of this study was to test the feasibility of telepsychology support for patients with severe cardiorespiratory disease and their caregivers. A secondary objective was to explore pre-post relationships between patients' and caregivers' clinical measures.

**Methods:**

A telehealth program incorporating telepsychology support, i.e., an “on-demand” phone service with a psychologist, was provided to consecutive cardiorespiratory patients at discharge from inpatient rehabilitation and to their caregivers. At the start and end of the 1-year program, participants were interviewed “face-to-face,” and their anxiety/depression level, patients' quality of life (MRF-28, SF-36, and MQOL), and caregivers' (*n* = 18) family strain (FSQ) and needs (CNA) were assessed: we analyzed the correlations and evaluated customer satisfaction.

**Results:**

Of 80 eligible individuals, 40 took part in this study: 22 patients (FVC = 39 ± 14%; EF = 39 ± 13%) and 18 caregivers. Eleven (28%, 6 patients and 5 caregivers) requested tele-psychological support, resulting in 51 consultations focused on anxiety, difficulty in patient management, worry about the patient's emotional state, and need for emotional support; 3 participants underwent a tailored psychotherapy program. All participants expressed high satisfaction with the service. At enrolment, anxiety was less evident in patients (73% men) than in caregivers, while depressive symptoms were more evident (6.5 ± 3.1), and correlated with MRF-28 and MQOL. Caregivers' (94% women) FSQ showed a “strongly recommended” need for support; at enrolment, high levels of anxiety/depression were correlated with high FSQ (for both, *p* < 0.05); depressive symptoms correlated negatively with age (*p* = 0.025) and positively with emotional needs (*p* = 0.025); anxiety was positively correlated with education level (*p* = 0.048). At follow-up, patients' perception of support (*n* = 13/22) tended to increase (*p* = 0.089), while caregivers' strain (*n* = 10/18) tended to decline (to within the “range of attention”). At enrolment, caregivers' anxiety/depression and strain correlated with patients' quality of life (for both; *p* < 0.05). At follow-up, caregivers' strain correlated with patients' quality of life (*p* = 0.028) and cognitive performance (*p* = 0.048).

**Conclusion:**

Telepsychology support associated with a telehealth service is feasible and satisfying for both participants and psychological management. A suitable support program can benefit both patients and caregivers, particularly those at higher risk of depressive symptoms (younger caregivers) and anxiety (all caregivers).

## Introduction

In recent years, there has been an increased focus on the management of cardiorespiratory diseases from a palliative point of view. Patients with advanced Chronic Obstructive Pulmonary Disease (COPD) and Chronic Heart Failure (CHF) require a specific dedicated approach due to their frequently impaired health status (Janssen et al., 2011) and uncertain prognosis with variable disease progression over time. Previous studies suggest that the health status of these patients may be even more impaired than that of patients with incurable cancer (Gore et al., 2000; O'Leary et al., 2009). Early identification of the clinical correlates of impaired health status can allow clinicians to modulate the therapeutic plan to stabilize the disease-specific health status and intervene more effectively through a better understanding of patients' needs (Gardiner et al., 2010; Gardener et al., 2018).

In a previous study (Vitacca et al., 2019), we showed the efficacy of a post-discharge telenursing program that offered patients with advanced COPD and CHF the availability of a specialist second opinion. It helped to satisfy patients' need to understand and manage better their symptoms and medication, reduce hospitalizations, support a healthy lifestyle, and enable prompt intervention where necessary. Access to the service and navigating it was easy, and patients could discuss end-of-life planning. Nevertheless, several signs emerged of diminished health status (general and disease-specific) in these patients. They included important psychological features such as anxiety, depression, and the ability to cope with feelings/worry and to try to live positively with the disease, think about the future, and support the family's needs (Gardener et al., 2018). It is well-known that symptoms of anxiety, depression, and care dependency are correlates of general and disease-specific health status in advanced COPD and CHF (Yohannes, 2007; Janssen et al., 2011; von Leupoldt and Kenn, 2013), and the psychological aspect plays an important role in the course and management of disease, not only for patients but also for their caregivers. Functional and emotional impairment has significant consequences for patients and their families: dependency on caregivers may lead to frustration, depression, and social isolation, increasing the burden on caregivers and their psychological sufferance (Fitzsimons et al., 2007).

For this reason, primary aim of this study was to test the feasibility of a model of telepsychology support offered on-demand to patients with advanced COPD and CHF and their caregivers. As a secondary aim, we wished to explore the changes at follow-up in all participants and in the patient-caregiver relationship through scales administered pre-post intervention by the psychologist assessing the self-reported quality of life, anxiety and depression symptoms, caregiver strain, and needs.

## Methods

### Study design

This was a prospective longitudinal study on the feasibility of a telemedicine service making psychologist support available for adult patients with severe cardiorespiratory at an advanced stage of disease following discharge from the hospital. The psychologist support service approached the “Nuove Reti Sanitarie” telehealth program managed by the Maugeri Center for Telehealth and Telecare (MCTT) (Scalvini et al., 2018). Patients were consecutively enrolled in the program following their discharge from the Rehabilitation Unit of the Istituti Clinici Scientifici Maugeri, IRCCS, Institute of Lumezzane (BS), Italy, where they had undergone a period of standard rehabilitation between October 2017 and May 2019. A previous report on the MCTT service focused on monitoring palliative care and including a few patients with COPD as a life-limiting disease showed its feasibility (Vitacca et al., 2019).

### Eligibility criteria: Patients and caregivers

Clinical criteria for patients' eligibility for the “Nuove Reti Sanitarie” telehealth program were as follows:

1. Patients with cardiac problems, NYHA class II-IV, heart failure or ventricular dysfunction, and ejection fraction <50% and at least one admission to the hospital in the previous 6 months;
2. Patients with respiratory problems, GOLD III-IV, and on long-term therapy with oxygen for at least 3 months.

Exclusion criteria were as follows: patients unable to be discharged home from the hospital; refusal to participate; and cognitive decline (MMSE <20).

At the time of the patient's admission to the program, the psychologist contacted their caregivers and proposed to them the same service of psychological support “on-demand.” Therefore, based on whether they agreed or declined, the study participants could be patients with their caregivers, or patients (or caregivers) separately. This study was approved by the institutional Ethics Committee (CE2108, 11 July 2017). All participants gave their signed informed consent to participate in the study.

### Intervention

At discharge from the hospital, patients were enrolled in “Nuove Reti Sanitarie,” which is a 6-month tele-assistance multidisciplinary support program designed to clinically follow patients at home and monitor their clinical problems, through telephone consultations managed by a nurse tutor. Patients can call the nurse if in clinical need (the service is available 24/7). Details on the tele-assistance are reported elsewhere (Scalvini et al., 2018; Vitacca et al., 2019). To better manage the patient, the MCTT program envisages the psychologist's collaboration with the nurse tutor and, if necessary, the clinician. The nurse tutor can also call on the psychologist to intervene in managing the patient's problems. Moreover, GPs are aware of the MCTT service and can be contacted by the psychologist, in particularly serious cases. Besides the patients, the psychologist also considers the patient's caregiver as a frail individual needing support when the patient is discharged home.

In this study, featuring the addition of telepsychology support directly to the participants for 1 year, at the time of the patient's discharge, the psychologist conducted an in-hospital “face-to-face” interview only with those patients/caregivers who adhered to the project. The aim was to collect clinical information and explain the modality of interaction with the telepsychology service. Tests assessing anxiety, depression, and quality of life were also administered. If warranted, the psychologist could suggest a tailored support program (clinical intervention).

Once at home, all participants could, if they needed any psychological help, call the psychologist. The psychologist was available to receive calls at all times (24/7). Such “on-demand” calls were independent of the scheduled phone calls performed by the nurse tutor in MCTT. The phone appointment was agreed on according to the psychologist's full availability and the patient's or caregiver's preferences. To reduce the sense of isolation and increase the perceived support, caregivers were also offered the possibility to join a monthly help group, in order to share experiences with others in a similar situation.

The duration of the psychology service was 1 year from the start (T0) for both patients and caregivers. All participants were reassessed at the end of the program (T1) in the hospital by the same psychologist using the same tools/assessments performed at T0.

### Feasibility

We evaluated the feasibility of the service in terms of participants' satisfaction. Customer satisfaction was assessed at the end of the program. We recorded the percentage of patients who agreed to do the program and their satisfaction, at the end of the program, with the service overall and with the psychologist's support (score 1= poor; 2= scarce; 3= good, and 4 = great).

### Outcome measures for patients and caregivers

The following questionnaires were administered to the patients:

- *Mini-Mental State Examination (MMSE)* (Folstein et al., 1975): a 30-item questionnaire to assess the cognitive status, with a score of <24 identifying cognitive deficit.
- *Anxiety-Depression Short Scale (AD-R)* (Moroni et al., 2006): to evaluate the presence of anxiety/depression symptoms, especially in people with cardiorespiratory diseases. It consists of 15 items testing depression (cutoff >7 for men and >9 for women) and 10 items testing anxiety (cutoff >22 for men, >25 for women): the higher the score in both dimensions, the greater the presence of significant symptoms.
- *Short Form Health Status (SF-36)* (Ware et al., 1993): a 36-item patient-reported survey of patient global health divided into eight domains, namely, physical functioning, physical role limitations, bodily pain, general health, vitality, social functioning, emotional role limitations, and mental health. For each domain, scores range from 0 (worst) to 100 (best); a physical and mental component summary is provided and scores range from 0 (worst) to 100 (best).
- *The Maugeri Respiratory Failure questionnaire (MRF-28)* (Vidotto et al., 2007): a 28-item disease-specific measure of health-related quality of life for patients with chronic respiratory failure; the higher the score, the more compromised the QoL. Two indexes can be found: disability perception and impairment in daily activities.
- *McGill Quality of Life, Italian version (MQOL-It)* (Sguazzin et al., 2010): a 16-item questionnaire to assess the quality of life in palliative care; items are rated on a numeral scale from 0 to 10, with a verbal anchor at the beginning and end of each visual scale. It has 5 domains, namely, physical symptoms, psychological wellbeing, existential area, support, and comparison with the health status of the previous year, and each domain scored from 0 (worst) to 10 (best) with a total score (0–10), which is the mean of the 5 subscales.

The following tests were administered to caregivers:

- *Anxiety-Depression Short Scale (AD-R):* as for patients (Moroni et al., 2006).
- *Family Strain Questionnaire-SF (FSQ-SF)* (Rossi Ferrario et al., 2001; Vidotto et al., 2010): a tool to assess the severity of burden in family caregivers; it consists of 30 dichotomous items, the higher the score, the more severe the strain.
- *Caregiver Needs Assessment (CNA)* (Moroni et al., 2008): to assess caregiver perceived needs related to assistance; it consists of 17 items referring to emotional needs, functional needs, cognitive-behavioral aspects, relational aspects, social needs, and spiritual ones. Each item is scored on a 4-point Likert scale from 0 (no need) to 3 (much need), giving a maximum total score of 51; two component summaries can be calculated: information/communication needs and need for emotional and social support.

### Statistics

Data were analyzed using Graph Pad-Prism software (version 8 for Windows, GraphPad Software, La Jolla California USA, www.graphpad.com) and expressed as mean ± standard deviation (SD). The normality of data was tested using the Shapiro-Wilk test. A paired *T*-test was performed to evaluate pre-post measures in both patients and caregivers with reevaluation at follow-up. Correlation analysis was conducted using the Pearson's test. Values of *p* < 0.05 were considered significant.

## Results

### Feasibility

**Figure 1:**
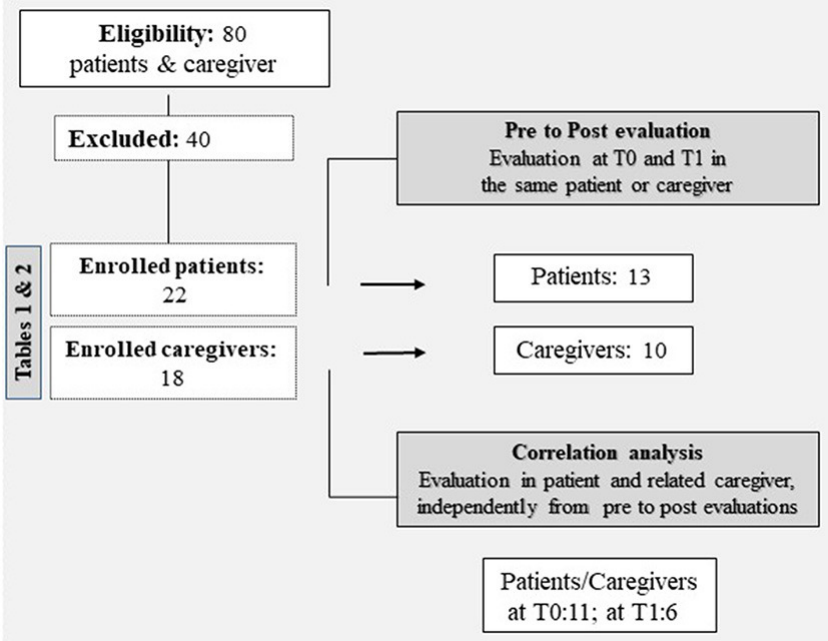
Flowchart of this study.

In this feasibility study, our team implemented the MCTT service with a telepsychology format, dependent on the MCTT for the patient's clinical care but independent as regards the management of the calls between the psychologist and participants. Telepsychology support was proposed to 80 individuals (patients and caregivers): 40 accepted and were eligible (50%); the 40 who declined did not give a reason (Figure 1).

Among the 40 participants who agreed to take part in the program, 22 were patients (Table 1) and 18 caregivers (Table 2); the vast majority (98%) came from the province of Brescia.

**Table 1:** Clinical characteristics and evaluations in patients at admission (T0) to the telepsychology service.

**Patients at T0 (*n* = 22)**	**Mean ±sd**
Gender, M/F	16/6
Age, years	71.6 ± 5.8
Education, years	7.1 ± 2.0
MMSE, score	27.0 ± 1.9
CIRS, score	1.9 ± 0.4
FEV1^*^, % pred	39.3 ± 14.5
EF^**^%	39.4 ± 13.2
Oxygen therapy, y/n	17/5
Anxiety scale, score	16.5 ± 3.4
Percentile	34.3 ± 22.6
**Depression scale, score**	6.5 ± 3.1
Percentile	70.9 ± 22.8
**SF-36 ISF index, score**	29.1 ± 6.6
Physical activity	38.2 ± 27.0
Physical limitation	9.1 ± 22.6
Physical pain	61.7 ± 27.1
Health	28.9 ± 18.2
**SF-36 ISM index, score**	44.9 ± 10.2
Vitality	49.3 ± 17.1
Social activity	53.2 ± 22.2
Emotional limitation	49.9 ± 42.1
Mental health	61.1 ± 18.5
**Total MRF-28, score**	12.9 ± 0.6.8
MRF-28-AQ	6.3 ± 3.8
MRF-28-DP	6.7 ± 3.6
MRF-28%	49.7 ± 25.1
**Global MQOL, score**	7.5 ± 1.4
Physical symptoms	7.3 ± 2.0
Psychological wellbeing	6.8 ± 1.8
Esistential area	7.2 ± 1.9
Support	8.1 ± 1.8

MMSE, Mini-Mental State Examination; CIRS, Cumulative Illness Rating Scale; FEV1, forced expiratory volume in 1 second; EF, Ejection fraction; SF-36, Short Form Health Status; MRF-28, Maugeri Respiratory Failure questionnaire; MQOL, McGill's Quality of Life questionnaire.

* Data available in 17 COPD patients (main diagnosis),

** available in 5 cardiac patients (main diagnosis).

6/22 Patients were cardiorespiratory patients, having both diseases.

**Table 2:** Clinical characteristics and evaluations in caregivers at admission (T0) to the telepsychology service.

**Caregivers at T0 (*n* = 18)**	
Gender (M/F), number	1/17
Age, years	64.9 ± 11.01
Education, years	8.7 ± 4.4
Anxiety scale, score	18.9 ± 7.1
Percentile	41.2 ± 31.8
Depression scale, score	3.7 ± 2.7
Percentile	39.2 ± 24.4
Total CNA, score	30.0 ± 10.5
CNA-emotion	13.2 ± 6.8
CNA-information	16.8 ± 4.9
FSQ, score	14.6 ± 7.7

CNA, Caregiver Needs Assessment; FSQ, Family Strain Questionnaire.

All patients had a visit with the psychologist during their in-hospital stay. At the time of patients' enrolment in the program (T0), their caregivers underwent a psychological interview. The psychological tests showed 2 out of 18 caregivers to be “at risk,” but they declined the psychologist's offer of a structured psychological program and opted instead for telepsychology support (in which they could call her during the year if in need).

The 18 caregivers who completed the psychology assessment were all relatives of the patient, without home help, and living with the patient, except for two. Out of 18, 11 caregivers participated in this study together with the patient, while 7 caregivers took part alone without the patient.

During the study period, 51 psychological consultations took place through phone call with 11 participants (28% of total participants), of whom 6 were patients and 5 were caregivers. The reasons for requesting a psychological consultation varied, but the interventions focused mainly on the following:

- Anxiety attacks.
- Depressive symptoms.
- Emotional problems/demoralization (fear of worsening).
- Relational problems.
- Difficulties in the patients' management.
- Discussing end-of-life topics.
- Fear of loss and grief.
- Help in the reorganization of daily activities.
- Support after re-exacerbation.

“Face-to-face” psychological support was provided to one patient, both during hospitalization and after enrolment in the program, with improvement. For 3 of the 6 patients who contacted the psychologist, a significant decrease in anxiety (a total reduction of anxiety attacks and improvements in the anxiety score at the end of the program) and depression level was reported. For one caregiver, a psychotherapy program took place “face to face” due to privacy problems at home, with great. Only 3 of the caregivers had previous experience with a psychologist: one for cognitive impairment of a relative, one for a daughter, and one for a son (reporting a negative experience).

Unfortunately, the monthly caregiver help group did not get underway due to a limited number of participants. The main reason was not lack of interest (several manifested interest) but the distance from the hospital and the lack of help with the patient's assistance.

All participants expressed a high level of satisfaction with the service of the Telehealth and Telecare program (MCTT) reporting it to be very useful in reducing the sense of abandonment and providing a sense of security and ease of utilization. All participants who had contact with the psychologist reported satisfaction with her service. All participants, both those who contacted the psychologist and those who did not, described the psychology service as important and useful and said they would recommend it.

### Patients and caregivers: Changes from enrolment to follow-up

Clinical and anthropometric characteristics of the 22 patients at T0 are reported in Table 1. Patients were mainly men (73%) and aged 63–87 years (Table 1).

The mean anxiety percentile was 34.3 ± 22.6, which is on average high but in the range of normality; only one patient had pathological values, and two were borderline. The mean depressive symptom percentile score was 70.9 ± 22.8, and 7 patients (5 out 7 were men) had scores above the significance level. The SF36 ISF index (physical index) mean score was 29.1 ± 6.6, while ISM (mental index) was 44.91 ± 10.19, showing a better mental health quality of life than a physical one, but both were below 50, i.e., below the norm. The MRF-28 mean score was 12.9 ± 0.6.8, and the MQOL mean total score was 7.5 ± 1.4, with psychological wellbeing as the lowest subscore. At T0, depression was positively related to MRF-28 score (*r* = 0.7557, *p* = 0.007), in particular to the perceived disability index (*r* = 0.9329, *p* = <0.0001); MQOL was negatively correlated with depression (*r* = −0.8680, *p* = <0.001), i.e., the lower the perceived quality of life, the higher the level of depression. MQOL was also negatively related to MRF-28, in particular to the perception of disability index (*r* = −0.7766, *p* = < 0.001). Almost all patients reported worrying about their illness worsening and limitations in activities, with a reactive state of demoralization and fear of symptoms; many patients (*n* = 12) reported more social isolation.

The clinical and anthropometric characteristics of the 18 caregivers at T0 are reported in Table 2.

Caregivers were mainly women (94%) and spouses, aged on average 65 years. The mean anxiety percentile was 41.2 ± 31.8 (i.e., in the range of normality), although 5 had pathological values. Caregiver anxiety positively correlated with education level (*r* = 0.606, *p* = 0.048). The mean depressive symptom percentile score was 39.2 ± 24.4, which is in the range of normality; only one participant had a pathological value. Depression symptoms were correlated with age (*r* = −0.665; *p* = 0.025) suggesting that younger caregivers experienced more depressive symptoms and anxiety (r = 0.713, *p* = 0.014). Depression was positively correlated with the emotional section of the Caregiver Needs Assessment (*r* = 0.666; *p* = 0.025): the more depressive symptoms were present, the more emotive needs caregivers experienced. Almost all caregivers reported worries about illness worsening and possible difficulties in the patient's clinical and emotional management; some of them reported more social isolation (*n* = 8).

At admission (T0), total CNA was 30.0 ± 10.5 points indicating moderate-high needs. FSQ score was 14.6 ± 7.7, a value indicating that psychological support is “strongly recommended.” At T0, caregivers with more anxiety and depression symptoms also had more burden/strain (*r* = 0.6056, *p* = 0.048 and *r* = 0.7958, *p* = 0.003, respectively). FSQ and CNA were positively correlated (*r* = 0.8062, *p* = 0.003), especially regarding the needs for social and emotional support: caregivers experiencing more strain recognized their need for support.

At follow-up (T1), there was a lower number of participants because some patients died or were admitted to other hospitals and their caregivers did not return to our Institute for the follow-up assessment. Out of 22, 13 patients were revisited in the hospital by the same psychologist. Evaluations were substantially unchanged, but the perception of support tended to increase (MQOL support: from 8.5 ± 1.3 to 7.7 ± 1.3, *p* = 0.089). At the same time, in 10 out of 18 caregivers, the family strain showed a trend to decrease (FSQ: from 14.6 ± 7.7 to 11.0 ± 6.0, *p* = 0.07) improving from “strongly recommended” support to within the “range of attention.” Levels of anxiety and depression were substantially unvaried in all caregivers, except for two who asked for help, and whose anxiety levels decreased. In general, caregivers perceived more anxiety, while patients experienced more depressive symptoms.

### Relation between patient and caregiver

At enrollment (T0), for patients enrolled together with their caregiver (*n* = 11), the patient's quality of life, especially the index of difficulties in daily activities (MRF-28 AQ), positively correlated with the caregiver's anxiety (*r* = 0.675, *p* = 0.023), depression (*r* = 0.840, *p* = 0.001), and strain (FSQ, *r* = 0.646, *p* = 0.032). The patient's MRF-28 total score also correlated with the caregiver's depression (*r* = 0.733, *p* = 0.010). At follow-up (T1), the caregiver's strain (FSQ) was significantly correlated with the patient's quality of life (*n* = 6, MRF-28 AQ: *r* = 0.861, *p* = 0.028) and cognitive performance (*n* = 6, MMSE: *r* = −0.882; *p* = 0.048).

At the end of the 1-year program, the psychologist reinterviewed all participants and readministered tests. She reported to the director of the MCTT service on the experience and planned, as an ongoing strategy, the stable inclusion of the psychologist in the MCTT, with the modalities of support and relative costs.

## Discussion

Our study confirmed the acceptability, feasibility, and satisfaction regarding a tele-psychological support service provided “on-demand” for patients with advanced cardiorespiratory diseases (clinically followed by a nurse tutor as regards their palliative needs) and their caregivers. The assessment of quality of life, anxiety, and depression symptoms in patients with severe cardiorespiratory in relation to the strain and needs of caregivers, before and after the 1-year psychology support service, confirmed the need for a dedicated figure to provide psychological support in conjunction with the MCTT team to optimize interventions.

Based on the previous literature on palliative care in patients with severe cardiorespiratory and our previous experience in supporting respiratory patients' clinical needs (Vitacca et al., 2019) at home, through the Telehealth and Telecare service (Scalvini et al., 2018), we opened this study also to caregivers to alleviate the family/caregiver strain and improve their QoL.

The availability of psychological support was agreed to by 50% and in the event requested by 28% of participants; it proved feasible and, according to what we observed, suitable psychological support could be the goal of a future study both for patients and caregivers. This is especially true for young caregivers, who could be more at risk of developing depressive symptoms, but also for all caregivers who have anxiety symptoms and high strain, or issues to discuss regarding end-of-life management.

Nevertheless, technology, age, and cultural barriers remain a restraint for elderly participants, who were not so familiar with the use of video-call programs or other open-source technological tools (e.g., such as Teams, Meet, or Zoom). In fact, the main criticism received from some older people was that they would have preferred a *face-to-face* contact to a phone call.

In planning telepsychology support for caregivers, the psychologist encouraged active caregiver engagement (providing emotional validation and eliciting/reinforcing change talk) and sought to build connections among caregivers by proposing a help group for caregivers. However, although several participants were favorable to the idea, many unmet needs remain such as the problem of assistance and support for displacement to access such services. This was the reason why the elderly caregivers could not participate in the help group. The connection among caregivers remains an important point to implement in developing such a service. While telepsychology would be easy to implement for young people smart with technology, it remains a critical point to solve for older participants.

In this study, 11 participants asked for a psychological consultation for various reasons, such as anxiety attacks, anxiety, and depressive symptoms, worries about the patient's emotional state, need for emotional support, difficulties in patient management, fear of loss, and grief. Participants who received psychotherapy support benefitted from an increase in wellbeing. At follow-up, the family strain showed a trend to decrease, while patients' perception of support tended to increase. All participants reported high satisfaction with the service; in particular, they saw it as very useful in reducing the sense of abandonment and giving a sense of security, and they declared it was easy to utilize.

Customer satisfaction with the overall service and with the psychological support in particular confirmed a high/very high satisfaction in all cases.

Concerning the psychologist's experience with the service, it was very positive in which, during this program, she encountered a particular niche of people. Most of them lived in valleys surrounding Brescia that were isolated for very long, which contributed to creating a more reserved outlook in which the psychologist was seen as the profession that deals with severe psychiatric problems, with a subsequent fear of stigma. Of the participants eligible for the study, 50% agreed to participate and approximately one-third received support from the psychologist. This is a very good result for the cultural and age reasons mentioned earlier. Things are changing today, with more opportunities to have access to a psychologist without fear of stigma.

In light of these findings, the director of the MCTT is evaluating the stable inclusion of the psychologist in the MCTT as an ongoing strategy and its relative costs. The status of future reimbursement for telepsychology service delivery is uncertain but, given its potential to reduce barriers, it needs to be adequately evaluated.

Our study also confirms previous reports in the literature (Janssen et al., 2011) that patients with advanced COPD and CHF have an impaired health status, a clearly lower quality of life, and a high level of anxiety, but especially higher depression symptoms. Although it was suggested that female patients with COPD had higher levels of anxiety and depression symptoms (Di Marco et al., 2006), this study showed a prevalence of depressive symptoms in men. Depression is correlated with the patient's quality of life: we think that the closure of possibilities, such as the impairment in physical and daily activities, with the consequent state of more dependency and social isolation, may have led from reactive demoralization to a state of depression. In fact, patients reported worries about physical limitations, increased dependency, and fear of worsening.

However, the average palliative care quality of life (MQoL) was quite high. If compared with the general population (SF36), the quality of life is clearly lower, but the quality of life specifically related to the palliative context appears better. This seems to adequately capture the awareness of being in a state of impairment but of receiving adequate management for the end-of-life period. Caregivers had a higher level of anxiety than patients, but lower depressive symptoms; they in fact reported worries about the patient's worsening and fear of not being able to manage the patient's symptoms.

Moreover, anxiety correlated with education level, suggesting that a higher education level can lead to being more informed and aware. Anxiety may arise in caregivers who, afraid of seeing their beloved suffering, feel inadequate to manage the clinical and emotional problems and fear losing them. Our study also found that younger caregivers are at higher risk of developing depressive symptoms, which may be a reaction to social isolation and limitation in activities, and depression was related to more emotional needs. Thus, more attention should be paid to the younger caregiver in assessing emotional symptoms, needs, and early intervention. This study confirmed the presence of high strain and burden in caregivers of patients with COPD and CHF, as reported in the literature (Luttik et al., 2007; Janssen et al., 2012; Cedano et al., 2013; Malik et al., 2013; Miravitlles et al., 2015).

It also confirmed the relation between high family strain levels and depression and anxiety, as reported elsewhere (Saunders, 2008). Importantly, this study highlights that caregivers felt more anxiety, depression symptoms, and strain when patients reported a low quality of life. Thus, it seems important to early assess patients and offer appropriate support for their needs in order to preserve their QoL as much as possible and to monitor/support caregivers in order to maintain the wellbeing of both. It is important to offer adequate support early to caregivers to maintain an acceptable QoL and reduce anxiety/depressive symptoms, in order to help caregivers reduce their strain and have a good influence on patients (Janssen et al., 2012), creating a virtuous circle. Moreover, the correlation between the patient's cognitive status and caregiver strain confirmed that low cognitive performance in patients increases the difficulty for caregivers to manage the clinical and emotional situation, i.e., increases the caregiver burden.

Gardener et al. (2018) identified, in patients with COPD and their relatives, 13 domains of support needs in COPD that are valid other clinical conditions: understanding the disease, managing symptoms and medication, maintaining a healthy lifestyle, managing feelings and worries, living positively with the disease, thinking about the future (including end-of-life planning), limiting anxiety and depression, obtaining practical support, managing finance/work/housing, developing family and close relationships, social and recreational life, maintaining independence, and ease in navigating the service. This psychological service within MCTT aims to address many of these needs. Obviously, further development is necessary to try to be as inclusive as possible.

### Limitations

This study has some limitations. First, the sample of participants was limited, resulting in insufficient statistical power in the pre-post analysis. Second, the phone as a medium for communication meant that many body language indicators of active listening and eye contact were difficult for the psychologist to perceive; a video call would enhance nonverbal communication. A further limitation was the nonavailability of an in-house transportation service for participants to access the hospital.

## Conclusion

Our study suggests that telepsychology support associated with a telehealth and telecare service is feasible for clinicians and participants. Participants expressed high satisfaction with the service provided by the psychologist–it was particularly important in reducing the sense of abandonment and providing an easy way to solve needs. Based on these observations, a suitable supportive program should be the goal of future research, in seeking to early evaluate and respond to patients' and caregivers' needs. Improving the QoL is especially important for young caregivers who may be at more risk of developing depressive symptoms, but for all caregivers, it is important to reduce their anxiety symptoms and high level of strain.

## Data availability statement

The raw data supporting the conclusions of this article will be made available by the authors, without undue reservation.

## Ethics statement

The studies involving human participants were reviewed and approved by Istituti Clinici Scientifici Maugeri CE2018, 11 July 2017. The patients/participants provided their written informed consent to participate in this study.

## Author contributions

LG and MV designed this study, collected and evaluated data, and prepared and critically reviewed the manuscript. LC performed the literature search, statistical analysis, and prepared and critically reviewed the manuscript. IT and SS collected data and critically reviewed the manuscript. All authors contributed to the article and approved the submitted version.

## Funding

We remember Prof. Jean-Marie Tschopp^†^ who strongly believed in this project and thank Dr. Jean-Michel Salamin and the Ligue Pulmonaire du District de Sierre (no profit organization), Switzerland for their advice and financial support for this project (free donation). This study was supported by the Ricerca Corrente Funding scheme of the Ministry of Health, Italy.

## Conflict of interest

The authors declare that the research was conducted in the absence of any commercial or financial relationships that could be construed as a potential conflict of interest.

## Publisher's note

All claims expressed in this article are solely those of the authors and do not necessarily represent those of their affiliated organizations, or those of the publisher, the editors and the reviewers. Any product that may be evaluated in this article, or claim that may be made by its manufacturer, is not guaranteed or endorsed by the publisher.
